# Neuroimmune regulation of post-traumatic bone regeneration: focus on inflammatory switching and functional recovery

**DOI:** 10.3389/fimmu.2026.1862080

**Published:** 2026-07-20

**Authors:** Wen-jia Du, Li Yan, Jun-wen Liang, Yi-wei Zhao, Ming-chun Li, Li-qiang Pan, Xiang-dong Yun

**Affiliations:** 1 Department of Orthopedics, Lanzhou University Second Hospital, Lanzhou, Gansu, China; 2 Key Laboratory of Orthopedics of Gansu Province, Lanzhou, Gansu, China; 3 Lanzhou Modern Vocational, Lanzhou, Gansu, China

**Keywords:** bone regeneration, functional repair, inflammatory regulation, macrophage polarization, neuro-immune axis, wound healing

## Abstract

The regenerative repair after bone trauma is not merely an osteogenic process but a dynamic reconstruction involving the coordinated participation of multiple systems, including the nervous, immune, and vascular systems. In recent years, the regulatory role of the neuro-immune axis in bone regeneration has attracted increasing attention. Existing studies indicate that this axis may influence the quality of bone regeneration and functional recovery outcomes by modulating inflammation initiation, facilitating the transition from the inflammatory clearance phase to the reparative phase, and contributing to the remodeling of the local microenvironment. Specifically, neural signal-mediated regulation of early immune cell recruitment, macrophage polarization, and angiogenesis-osteogenesis coupling represents a critical upstream mechanism in post-traumatic bone regeneration. Conversely, an imbalance in the neuro-immune axis may be associated with adverse outcomes such as nonunion, chronic pain, and functional impairment. This article reviews the main mechanisms by which the post-traumatic neuro-immune axis regulates bone regeneration from four aspects: inflammation initiation, inflammation switching, microenvironment remodeling, and functional repair, and summarizes the research progress of related intervention strategies. Overall, targeting the neuro-immune axis may provide novel therapeutic strategies for promoting bone healing and improving functional recovery; however, most current evidence is derived from animal experiments, mechanistic studies, or early translational explorations, and its clinical value remains to be further validated.

## 1 Introduction

Post-traumatic bone regeneration is far more than the mere activation of osteoblasts and matrix mineralization; it is a spatiotemporally dynamic process involving the coordinated interplay of the nervous, immune, vascular, and skeletal systems (1, 2). Bone tissue is richly innervated and vascularized, and sensory and sympathetic nerves in the peripheral nervous system not only participate in bone development and homeostasis but may also play a crucial regulatory role in the repair process after injury (3, 4). Traditional bone repair research has primarily focused on osteoblasts, growth factors, and biomaterial properties, with relatively less attention paid to neural regulation and its intricate crosstalk with immune responses, thereby constraining our comprehension of the clinical phenomenon where “similar bone injuries lead to different healing outcomes” (5, 6).

In recent years, the neuro-immune axis has gradually emerged as an important perspective for explaining the heterogeneity of post-traumatic bone regeneration. This axis emphasizes that damage-associated molecular patterns (DAMPs) released after injury not only activate innate immune responses but also simultaneously stimulate sensory and sympathetic nerves innervating bone tissue, thereby regulating the recruitment, activation, and phenotypic polarization of local immune cells through neuropeptides, neurotransmitters, and related effector molecules (7). Therefore, the nervous system extends beyond its canonical role in injury perception and pain transmission; it may also influence the subsequent bone regeneration process by orchestrating inflammatory responses.

An increasing number of studies now suggest that the key to post-traumatic bone regeneration lies not in the presence or absence of inflammation, but in whether inflammation can efficiently shift from the clearance phase to the repair phase within an appropriate time window (8, 9). This “inflammatory switch” process may determine whether the local microenvironment remains in a pro-inflammatory and tissue-damaging state or shifts toward a pro-repair state conducive to angiogenesis, mesenchymal stem cell recruitment, and osteogenic differentiation (10, 11). The neuro-immune axis may be one of the key upstream regulatory systems influencing this temporal transition: on one hand, neural signals can affect the early initiation of inflammation and immune cell infiltration (12); on the other hand, they can also promote the orderly progression of bone regeneration into the repair phase by modulating macrophage functional status, inflammation resolution, and neurovascular coupling (13). Conversely, dysregulation of this axis may be associated with adverse outcomes such as persistent inflammation, nonunion, chronic pain, and poor functional recovery (14).

Based on the above background, this article will review the main regulatory mechanisms of the neuro-immune axis from four aspects: inflammation initiation, inflammatory switch, microenvironment remodeling, and functional repair in post-traumatic bone regeneration, and further discuss the regenerative disorders that may result from its imbalance, as well as related intervention strategies.

## 2 The inflammatory switching framework of post-traumatic bone regeneration: from osteoimmunity to the neuro-immune axis

### 2.1 Stages of bone regeneration and the inflammatory time sequence

Post-traumatic bone regeneration is a highly ordered and dynamically advancing process, typically summarized into three consecutive stages: the inflammatory phase, the repair phase, and the remodeling phase (15, 16). Unlike traditional approaches that view bone healing solely from a histomorphological perspective, current research emphasizes that it is essentially a regenerative process tightly orchestrated by the inflammatory time sequence: early inflammation initiates clearance and recruitment programs, mid-phase inflammation subsides to create conditions for tissue reconstruction, and the later phase achieves structural optimization and functional recovery through osteoblast-osteoclast coupling (17, 18). During the inflammatory phase, vascular rupture after fracture forms a hematoma, and DAMPs are rapidly released, triggering an acute inflammatory response characterized by neutrophil and pro-inflammatory macrophage infiltration (19, 20). The primary functions of this phase are to clear necrotic tissue, limit infection, and establish the initial repair microenvironment, thereby recruiting cell populations necessary for subsequent repair via the release of inflammatory cytokines such as TNF-α, IL-1β, and IL-6 (21). If this process is insufficient, repair initiation signals are inadequate; if it is excessive or prolonged, it may lead to exacerbated tissue damage and delayed healing (22).

As inflammation gradually becomes controlled, bone regeneration enters the repair phase. Consequently, the inflammatory response transitions from a pro-inflammatory state to a pro-repair state, with macrophage phenotypes shifting from a predominantly M1 to an M2 phenotype. Angiogenesis increases, and mesenchymal stem cells and osteoprogenitor cells are recruited, differentiating into chondroblasts and osteoblasts, gradually forming cartilage callus and woven bone (23, 24). The core of this phase is not the complete disappearance of inflammation, but the formation of a synergistic relationship between inflammation-related programs and vascularization, cell recruitment, and osteogenic activity that is more conducive to repair. In the remodeling phase, the early-formed woven bone is gradually replaced by lamellar bone with a more ordered structure and better mechanical properties, further restoring the morphology and biomechanical function of bone tissue (25). This process relies on dynamic coupling between osteoblasts and osteoclasts and is also influenced by factors such as residual local inflammation, innervation status, and mechanical load (26, 27). Therefore, from the inflammatory phase to the repair phase and then to the remodeling phase, it is not a simple temporal progression but a continuous regenerative process driven jointly by the inflammatory time sequence, tissue reconstruction, and bone remodeling.

### 2.2 The core role of immune cells in bone regeneration

The success of bone regeneration largely depends on the quality of the local immune microenvironment, with immune cells serving as the primary orchestrators of this microenvironment. They not only participate in the initiation of inflammation after injury but also directly influence angiogenesis, mesenchymal stem cell homing, and osteoblast/osteoclast balance by secreting cytokines, chemokines, and growth factors (28, 29). Therefore, bone regeneration is not solely dominated by the bone cell system but is a regenerative process “directed” by the participation of immune cells.

Macrophages are one of the important effector cells. It should be emphasized that the M1-like/M2-like classification is only used to summarize the main functional tendencies of macrophages at different repair stages and does not represent a strictly dichotomous fixed phenotype in the trauma microenvironment. In actual bone repair processes, macrophages are more likely to exhibit a continuous spectrum and dynamic transitional state, with their functions jointly influenced by the injury time window, fracture site, infection status, material interface, and host baseline state. Therefore, the M1-like state should not be simply understood as “harmful,” nor should the M2-like state be absolutely equated with “beneficial”; both need to function in the appropriate temporal and spatial context.

In the early stage of trauma, M1-like macrophages primarily undertake the functions of clearing necrotic tissue, limiting pathogen spread, and initiating necessary inflammatory responses (30). Subsequently, if the healing process progresses smoothly, macrophages gradually shift towards an M2-like pro-repair phenotype, secreting anti-inflammatory and pro-repair factors such as TGF-β, IL-10, and Vascular Endothelial Growth Factor (VEGF), thereby promoting angiogenesis, osteogenic differentiation of mesenchymal stem cells, and matrix deposition (31, 32). Consequently, the macrophage transition from a pro-inflammatory/clearance state to an anti-inflammatory/pro-repair state represents a pivotal biological event marking the shift from the inflammatory to the repair phase, serving as a key indicator of successful bone regeneration. In addition to macrophages, T lymphocytes also participate in the regulation of bone regeneration. Regulatory T cells (Tregs) typically help limit excessive inflammation and support osteogenesis by secreting repair-related factors, whereas Th17 cells and their characteristic cytokine IL-17 can, under certain conditions, exacerbate inflammation and promote osteoclast activity (33). This difference suggests that adaptive immunity does not unidirectionally promote or inhibit bone repair but exerts bidirectional regulatory effects at different stages and in different microenvironments. Neutrophils, as the first innate immune cells to arrive at the injury site, are primarily responsible for acute phase defense and inflammatory amplification (34). A moderate neutrophil response aids in clearing necrotic tissue and initiating repair, but if persistently activated, they can aggravate local tissue damage by releasing reactive oxygen species (ROS) and proteases, destroying new blood vessels and the extracellular matrix, thereby hindering repair (35). Therefore, ideal bone regeneration does not require complete suppression of the immune response but rather requires different immune cells to participate at the correct time and with appropriate intensity. Consequently, immune regulation in bone regeneration should be understood as a multi-cell, multi-stage synergistic process, not something that can be explained by a single immune cell type or a single phenotypic change.

### 2.3 From inflammatory “initiation” to inflammatory “switching”

In the process of bone regeneration, the key factor affecting healing quality is not the presence or absence of an inflammatory response, but whether inflammation can switch from a clearance program to a repair program within an appropriate time window (36). Early inflammation aids in necrotic tissue clearance, establishment of chemotactic signals, and recruitment of repair cells; however, if the pro-inflammatory response persists, leading to a long-term local state of high TNF-α, IL-1β, IL-6, and ROS load, it may inhibit the osteogenic differentiation of mesenchymal stem cells, promote osteoclast activity, and increase the risk of delayed healing or non-union (37–39). Therefore, inflammatory switching should be understood as a dynamic temporal transition, rather than a simple fluctuation in inflammatory intensity.

In this process, the functional shift of macrophages from an M1-like pro-inflammatory/clearance state to an M2-like anti-inflammatory/pro-repair state is a prominent quantifiable hallmark, but it is not the sole indicator (40, 41). Inflammatory switching also includes multiple aspects such as timely clearance of neutrophils, a decrease in pro-inflammatory mediators, an increase in pro-resolving mediators, enhanced angiogenesis, and the gradual dominance of osteogenesis-related signals (42). Among these, the functional state of macrophages can be tracked through M1-like related indicators such as inducible nitric oxide synthase (iNOS), CD86, TNF-α, and IL-1β, as well as M2-like/pro-repair related indicators such as arginase-1 (Arg1), CD206, IL-10, and TGF-β. The relevant time windows and marker changes are shown in **Table 1**.

**Table 1:** Dynamic changes in macrophage M1/M2 markers during post-traumatic bone repair and their association with inflammatory switching.

Repair phase/time window	Major macrophage phenotype	Changes in M1 markers	Changes in M2/pro-repair markers	Main functional significance	Representative evidence	Reference
Early Inflammation Phase	M1-like macrophages dominant	Elevated levels of iNOS, CD86, TNF-α, and IL-1β	CD206, Arg1, IL-10, etc., relatively low or not yet dominant.	Clearance of necrotic tissue, initiation of acute inflammatory response, recruitment of repair-related cells.	Monocyte/macrophage migration and elevation of pro-inflammatory factors in early human fracture hematomas; early dynamic upregulation of inflammatory factors in animal models.	(103, 104)
Inflammation-to-Repair Transition Phase	M1 decreases, M2-like macrophages increase	Pro-inflammatory signals such as TNF-α, IL-1β, IL-6 gradually decrease.	Arg1, CD206, IL-10, TGF-β, etc., gradually increase.	Promotion of inflammation resolution, angiogenesis, MSC recruitment, and formation of a repair microenvironment.	In human clavicle fracture samples, M1 macrophages decrease over time, while M2 macrophages increase with repair progression, correlating with larger callus volume and shorter healing time.	(105)
Repair Phase (Callus Formation)	M2/pro-repair macrophages dominant	Pro-inflammatory markers further decrease or remain at low levels.	VEGF, TGF-β, BMP-2, IL-10, etc., enhanced.	Support angiogenesis, osteogenic differentiation, cartilage-to-bone callus conversion, and matrix deposition.	Mouse closed femoral fracture study shows M2 macrophages are dominant during the ossification phase, and IL-4/IL-13-induced M2 polarization enhances bone formation.	(106)
Remodeling Phase	Macrophage phenotype tends toward homeostasis or transitions to a tissue-resident/regulatory state.	Inflammatory markers remain at low levels	Gradual decline in repair-related signals, which continue to participate in homeostasis maintenance.	Involvement in osteoblast-osteoclast coupling, bone matrix remodeling, and long-term tissue homeostasis restoration.	Bone defect and fracture models suggest macrophage involvement in later-stage bone regeneration, neovascularization, and bone remodeling; M2 deficiency in osteoporosis/aging models correlates with impaired repair.	(107, 108)

**Figure 1:**
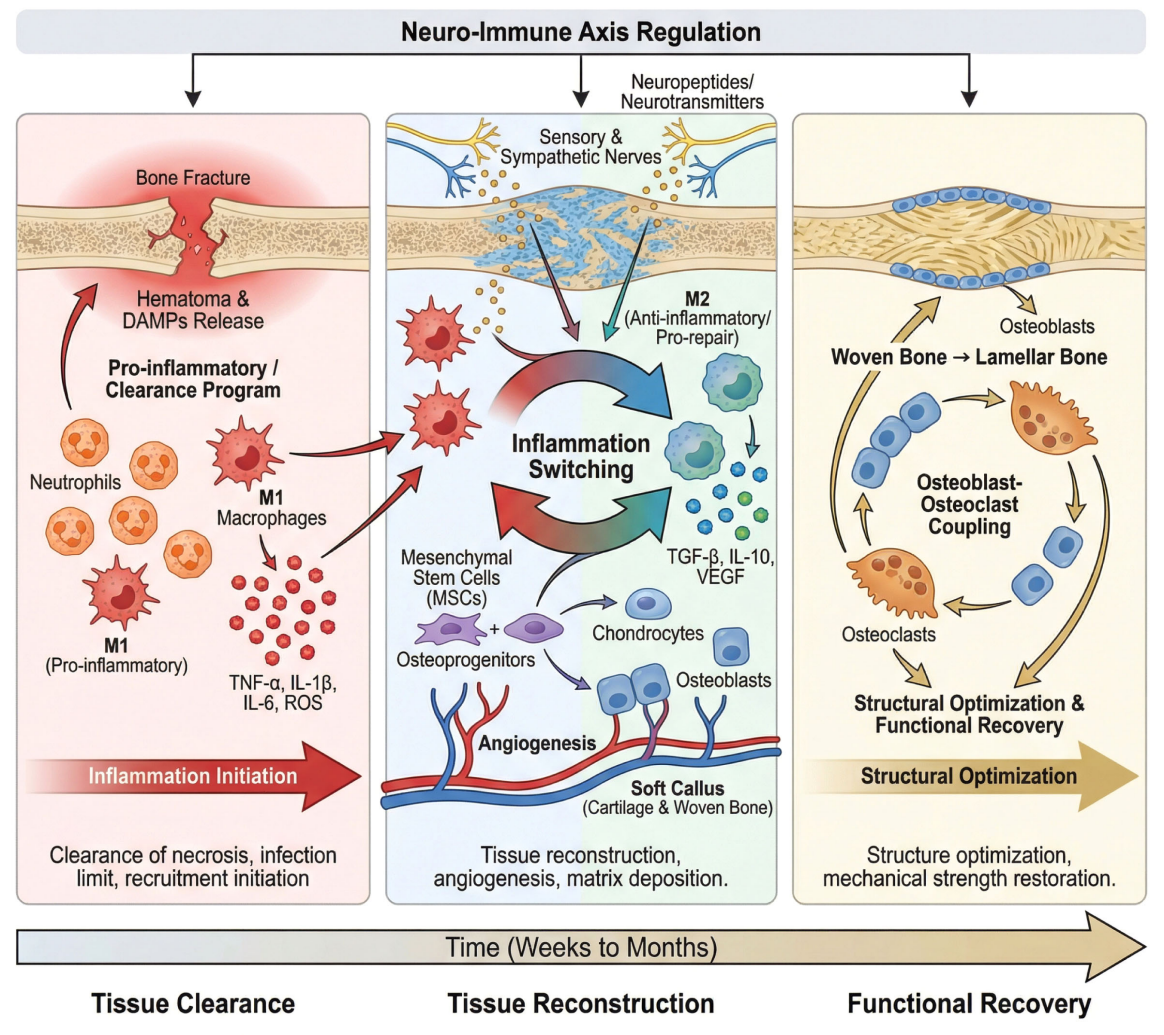
The stage-specific regulatory role of the neuro-immune axis during fracture repair. After fracture and hematoma formation, the release of DAMPs triggers an early pro-inflammatory/clearance program mediated by neutrophils and M1-like macrophages to accomplish necrotic tissue clearance and initiate repair recruitment. Subsequently, neuropeptides and neurotransmitters released by sensory nerves and sympathetic nerves orchestrate the transition of inflammation from the pro-inflammatory phase to the pro-repair phase, driving macrophage polarization toward an M2-like phenotype, and supporting mesenchymal stem cell recruitment, angiogenesis, cartilage and osteogenic differentiation, and cartilage callus formation. Finally, during osteoblast-osteoclast coupling and bone remodeling, woven bone is gradually transformed into lamellar bone, achieving structural optimization and functional recovery.

The neuro-immune axis may be one of the important regulatory systems affecting the temporal transition of inflammation. After injury, sensory nerves and sympathetic nerves can detect alterations in the local microenvironment and modulate immune cell recruitment, macrophage functional state, and local vascular responses through neuropeptides and neurotransmitters (43). Therefore, neural regulation is better understood as an upstream factor influencing the direction of inflammatory switching and the repair time window, rather than the sole mechanism determining the final outcome of bone healing (44). Based on this understanding, the focus of research on post-traumatic bone regeneration is shifting from simply modulating inflammatory intensity to regulating the timing, threshold, and direction of inflammatory switching, and the neuro-immune axis is the key entry point for understanding and intervening in this process (**Figure 1**).

## 3 Post-traumatic neural remodeling and its fundamental role in bone regeneration

### 3.1 Anatomical and functional zonation of bone innervation

Bone tissue is not merely a mechanical support structure but a living tissue with abundant innervation. Its neural sources primarily include sensory nerves and sympathetic nerves, which together form the anatomical basis of post-traumatic neuro-immune-bone regulation (45, 46). Sensory nerves are widely distributed in the periosteum, bone marrow cavity, and around trabeculae, primarily responsible for nociception. They modulate local inflammatory regulation, angiogenesis, and osteogenesis by releasing neuropeptides such as calcitonin gene-related peptide (CGRP) and substance P (SP) (47, 48). Therefore, the significance of sensory nerves in bone regeneration extends beyond pain conduction; they serve as an important source of local repair signals. In contrast, sympathetic nerves are mainly distributed along intraosseous blood vessels, releasing norepinephrine (NE) at their terminals, which plays a crucial role in regulating bone tissue blood perfusion, immune cell status, and bone metabolism balance (49). Moderate sympathetic activity helps maintain local homeostasis, but sustained or excessive sympathetic excitation may amplify inflammatory responses, promote osteoclast activity, and inhibit osteogenesis, thereby hindering bone repair (50, 51). From a functional zonation perspective, sensory nerves are more inclined toward “injury perception—neuropeptide release—repair promotion,” while sympathetic nerves are more involved in “blood flow regulation—immune modulation—bone metabolism balance” maintenance (43).

### 3.2 Post-traumatic nerve injury and adaptive remodeling

Fractures and related soft tissue injuries not only damage the bone structure itself but also often cause axonal damage to nerves innervating bone and surrounding tissues, triggering Wallerian degeneration. After nerve transection, distal axons and myelin sheaths disintegrate, with local debris clearance and Schwann cell responses forming the basis for subsequent regeneration (52, 53). For bone regeneration, this process represents not only a temporary interruption of neural conduction but also entails that local neuropeptide release, sensory feedback, and neuro-immune regulatory networks are compromised in the early phase, potentially affecting inflammatory switching and repair program initiation (54). As healing progresses into the repair phase, the damaged nervous system can exhibit adaptive remodeling, characterized by axonal sprouting, reinnervation, and functional neural network reconstruction (55). This process typically relies on the upregulation of neurotrophins such as nerve growth factor (NGF) and brain-derived neurotrophic factor and is closely coupled with local angiogenesis. Newly formed blood vessels not only provide oxygen and nutrients but also offer guidance and support for regenerating axons, thereby forming a mutually reinforcing process of neural regeneration and angiogenesis (56). Post-traumatic neural remodeling is not an incidental phenomenon independent of bone repair but a crucial component of the bone regeneration microenvironment reconstruction. The success or failure of reinnervation directly affects the quality of bone regeneration and subsequent functional recovery (57). Insufficient local neural remodeling may lead to decreased neuropeptide supply, persistent inflammatory responses, and abnormal pain recovery, increasing the risk of delayed union, nonunion, and chronic pain (58, 59). Conversely, appropriate reinnervation helps restore local blood flow regulation, optimize the immune microenvironment, and promote bone tissue reconstruction (60). Therefore, the adaptive remodeling of the post-traumatic nervous system constitutes a fundamental event in the bone regeneration process.

### 3.3 Pleiotropic functions of key neural signaling molecules in bone repair

During post-traumatic bone regeneration, multiple neural-derived molecules participate in the coordinated regulation of local inflammation, angiogenesis, and osteogenic activity, with CGRP, SP, and NE being the most representative (61). CGRP is an important neuropeptide derived from sensory nerves, possessing pro-angiogenic, pro-osteogenic, and immunomodulatory effects. On one hand, it promotes endothelial cell proliferation and angiogenesis, improving local blood supply; on the other hand, it directly acts on osteoblast-related cells, enhancing osteogenic differentiation and bone matrix deposition, and participates in regulating the functional status of immune cells such as macrophages, thereby facilitating the establishment of a favorable repair microenvironment (62, 63). CGRP can be regarded as an important bridge connecting sensory nerve activity and bone regeneration responses, and its deficiency often indicates impaired local neural regulation (64). SP, in contrast, is more prominently involved in the early initiation of inflammation. As another sensory neuropeptide, SP increases local vascular permeability and promotes immune cell recruitment to the injury site, thereby amplifying the necessary early inflammatory response (65). Furthermore, SP can act on mesenchymal stem cells and augment their reparative capacity, suggesting its bridging role between inflammation initiation and osteoprogenitor cell activation. The primary significance of SP lies not in maintaining long-term inflammation but in providing a sufficiently strong early initiation signal for the repair program (66).

**Figure 2:**
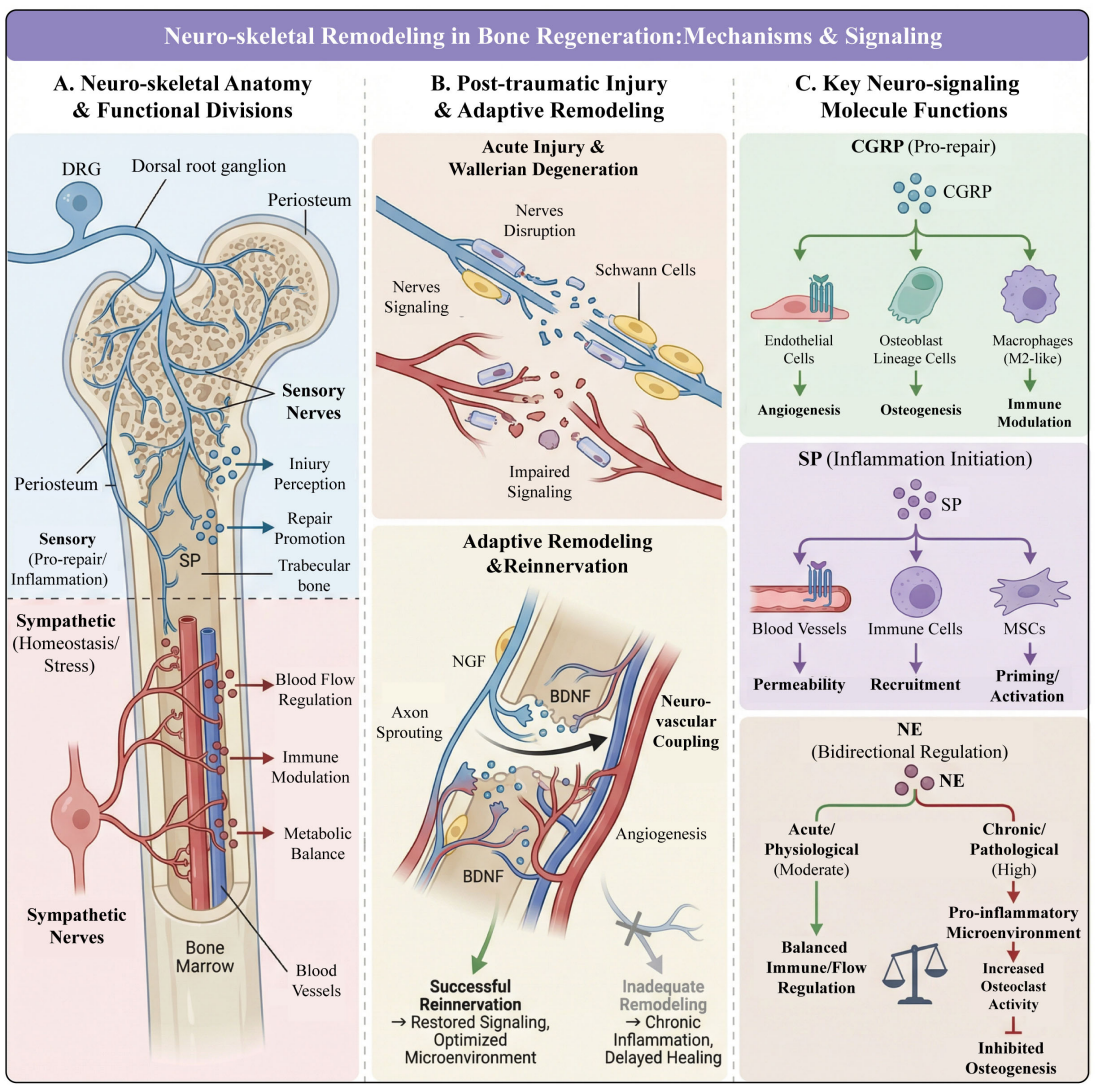
Mechanisms and signaling regulation of neuro-skeletal remodeling in bone regeneration.This figure summarizes the roles of sensory and sympathetic nerves in bone homeostasis, post-traumatic remodeling, and regeneration. **(A)** Sensory and sympathetic nerves in the periosteum, bone marrow, and trabecular bone regulate injury perception, repair promotion, blood flow, immune responses, and metabolic balance. **(B)** Post-traumatic nerve disruption and Wallerian degeneration impair local signaling, whereas adaptive reinnervation and neurovascular coupling help restore the microenvironment and support bone repair. **(C)** Neuro-signaling molecules, including CGRP, SP, and NE, regulate angiogenesis, osteogenesis, immune cell recruitment, and osteoclast activity, thereby influencing successful regeneration or delayed healing with chronic inflammation.

NE is the main neurotransmitter of the sympathetic nervous system, exhibiting a distinct biphasic regulatory effect on bone repair. In the early stress phase, NE can participate in the regulation of immune and blood flow responses; however, if the sympathetic nervous system is overactivated and NE levels remain chronically elevated, chronic elevation of NE tends to sustain a pro-inflammatory microenvironment, enhance osteoclast activity, and inhibit osteogenesis, ultimately impeding bone healing (67). Therefore, NE is not simply a harmful signal but a regulatory factor highly dependent on phase and intensity. From a translational perspective, suppressing the adverse effects of sustained NE elevation while preserving its physiological regulatory functions remains a key challenge for future neuroregulatory interventions (68). Overall, CGRP, SP, and NE represent three major neural signaling modalities: repair promotion, inflammation initiation, and stress-related biphasic regulation (**Figure 2**).

## 4 Neuro-immune axis regulates the initiation of inflammation after trauma

### 4.1 DAMPs release: a common activating signal for nerves and immunity

Fractures and related tissue damage can lead to cell rupture and matrix destruction, thereby releasing substantial quantities of endogenous DAMPs (69). These molecules constitute the initial “danger signals” for the initiation of post-traumatic inflammation and are also an important starting point for the co-activation of the nervous and immune systems (70). In the context of bone trauma, HMGB1, ATP, and heat shock proteins (HSPs) are representative DAMPs. On one hand, they can be recognized by pattern recognition receptors on immune cells such as macrophages, especially Toll-like receptors, thereby initiating innate immune responses and inducing the release of pro-inflammatory cytokines (71); on the other hand, they can also act on receptors on sensory nerve endings, such as P2X and TRPV1 channels, promoting nerve excitation and neurogenic inflammation (72). Therefore, the significance of DAMPs is not merely to “initiate inflammation,” but to establish the initial coupling state of the post-traumatic neuro-immune axis by simultaneously activating both the immune and sensory nervous systems. In other words, the initiation of inflammation after bone trauma is not a purely immune event, but a coordinated response between nerves and immunity mediated by DAMPs. It is also at this stage that the local microenvironment begins to determine whether the subsequent inflammatory response follows the trajectory of “appropriate clearance—timely switching” or towards excessive amplification and persistent dysregulation (73).

### 4.2 “Chemotactic” effects of sensory neuropeptides on early immune cell recruitment

Following the initial activation triggered by DAMPs, neuropeptides released by sensory nerves further participate in the recruitment of early immune cells, driving the inflammatory response to aggregate at the injury site (74). CGRP and SP are the most representative sensory nerve signaling molecules at this stage (75). Existing studies suggest that these neuropeptides can not only affect local vascular tone and permeability but also promote the migration and extravasation of immune cells to the injury site by acting on neutrophils, monocytes, and related endothelial cells (76, 77). Therefore, sensory nerves are not merely passive conduits for nociceptive signaling after trauma but directly participate in the early process of immune cell recruitment. Among them, SP is more inclined to amplify the early inflammatory response. It can increase local vascular permeability, promote the recruitment of leukocytes, especially neutrophils and monocytes, thereby enhancing the aggregation effect of inflammation at the injury site (78, 79). The role of CGRP is more regulatory: on one hand, it can affect immune cell extravasation by modulating local microvascular responses and endothelial status (80); on the other hand, its specific effects may be tissue- and phase-dependent (81). Overall, the main role of CGRP and SP at this stage is to provide conditions for chemotaxis and vascular extravasation for early immune cell infiltration, thereby promoting the rapid establishment of the post-traumatic inflammatory response.

### 4.3 Direct regulation of immune cell function by neural signals

In addition to influencing early recruitment, neural signals can also directly reshape the functional state of immune cells, thereby affecting the subsequent direction of the inflammatory response (82). CGRP is the most typical sensory nerve-derived regulatory factor. Current studies indicate that CGRP can inhibit the production of pro-inflammatory cytokines such as TNF-α and IL-12 by macrophages, while promoting the expression of anti-inflammatory/repair factors such as IL-10, thereby limiting excessive inflammation and driving the transition of macrophages towards an M2-like functional state (83). Therefore, CGRP not only possesses the attribute of a “sensory signal” during the initiation phase of inflammation but also plays a role in “inflammatory braking and functional reprogramming,” and can be regarded as an important neural regulatory factor that promotes the transition of inflammation from amplification to controlled resolution. In comparison, the sympathetic neurotransmitter NE exerts a more bidirectional and phase-dependent regulatory effect on immune cells (84, 85). Moderate NE signaling may mitigate non-specific immune attacks and maintain local homeostasis; however, under conditions of persistent stress or excessive sympathetic nerve activation, chronically elevated NE tends to disrupt normal immune balance, maintain an aberrant inflammatory state, and adversely affect subsequent repair (86). Therefore, the role of NE cannot be simply classified as pro-inflammatory or anti-inflammatory but should be understood as a dynamic regulatory signal dependent on concentration, exposure time, and microenvironmental context (87). In summary, the role of neural signals in the initiation of inflammation is not limited to “recruiting” immune cells but extends to “programming” their functional state. It is this continuous regulation, from recruitment to functional remodeling, that makes the neuro-immune axis a crucial upstream system determining the quality of post-traumatic inflammation and the direction of the subsequent response.

### 4.4 Neural regulation imbalance and pathological inflammation

**Figure 3:**
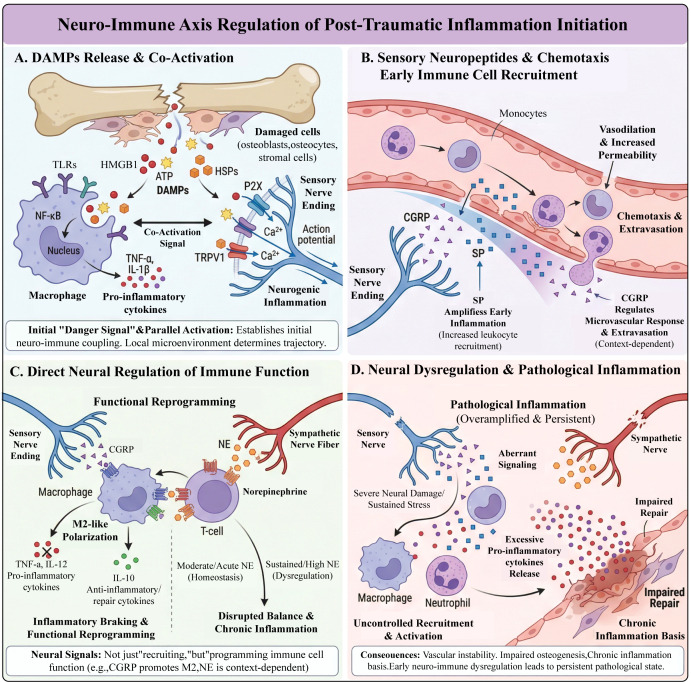
Framework of neuro-immune axis regulation during the initiation phase of post-traumatic inflammation. **(A)** After bone injury, damaged cells release DAMPs such as ATP, HMGB1, and HSPs, activating macrophages and sensory nerve endings, triggering NF-κB signaling, pro-inflammatory cytokine release, and neurogenic inflammation. **(B)** Sensory nerves release CGRP and SP, mediating vasodilation and increased vascular permeability, as well as facilitating the early chemotaxis and extravasation of immune cells such as monocytes. **(C)** Neural signals not only recruit immune cells but can also directly reshape their function; for example, CGRP promotes the polarization of macrophages toward an M2-like phenotype, while norepinephrine exerts context-dependent regulatory effects on T cell function and the overall inflammatory response. **(D)** Imbalanced neural regulation or persistent nerve damage can lead to excessive amplification of inflammation, abnormal immune cell recruitment, and impaired repair, ultimately resulting in a chronic pathological inflammatory state.

When trauma causes severe damage to the local neural network, or persistent stress leads to excessive sympathetic nerve excitation, the normal regulatory order of the neuro-immune axis is disrupted, and the inflammatory initiation process is more likely to shift from appropriate defense to pathological amplification (88). In this case, the release profile of neuropeptides and neurotransmitters becomes abnormal, the recruitment and early activation of immune cells lose temporal control, and the local inflammatory response can exhibit features of hyperactivity, prolongation, or dysregulation from the initiation stage (89). Therefore, neural regulation imbalance is not merely a concomitant phenomenon but an important driving factor for the formation of pathological inflammation. It is important to emphasize that the core issue is primarily the dysregulation of the inflammatory initiation program, not merely insufficient later-stage repair (90). That is to say, once the neuro-immune coupling becomes abnormal in the early stage, the trauma site is more prone to forming an excessively amplified inflammatory input, laying the foundation for subsequent persistent inflammation (91). At this point, vascular endothelial stability, the tolerance of osteogenic precursor cells, and the local repair signaling environment may all be disturbed in the early stage (92). Therefore, understanding the normal regulatory mechanisms of the neuro-immune axis during the inflammatory initiation phase not only helps explain the heterogeneity of post-traumatic inflammatory responses but also provides a theoretical basis for future interventions using neural regulation methods to reduce early inflammatory dysregulation (**Figure 3**).

## 5 The key role of the neuro-immune axis in reshaping the bone regeneration microenvironment

### 5.1 Core mechanisms driving macrophage polarization switching

In post-traumatic bone regeneration, the functional shift of macrophages from a pro-inflammatory clearance state to an anti-inflammatory/pro-repair state is a key execution step in inflammatory switching (93, 94). Crucially, this process does not merely equate to the “disappearance of M1-like and appearance of M2-like” phenotype, but rather a dynamic rearrangement of multiple transitional, mixed, and tissue-specific macrophage states across spatiotemporal dimensions. In other words, the M1-like/M2-like classification is primarily used to summarize functional tendencies and cannot replace the understanding of the continuous spectrum of states in the real traumatic microenvironment.

Sensory neuropeptides are important upstream signals regulating the functional state of macrophages. CGRP and vasoactive intestinal peptide (VIP) have both been reported to promote an anti-inflammatory/pro-repair functional shift. CGRP can inhibit the release of certain pro-inflammatory factors through its receptor-mediated signaling pathways and promote the expression of reparative factors such as IL-10, thereby facilitating the transition of macrophages towards a pro-repair state (89). VIP also exhibits similar anti-inflammatory and pro-repair effects, potentially participating in limiting excessive inflammation and establishing local repair programs (95). Therefore, sensory nerves not only transmit nociceptive signals but can also modulate macrophage functional reprogramming through neuropeptide signaling (96, 97).

In contrast, the influence of sympathetic nerve signals on macrophage function is more phase-dependent and context-dependent. In the early stage of inflammation, elevated NE may be associated with enhanced pro-inflammatory responses and, to some extent, support early clearance programs (98); whereas during the repair progression phase, sympathetic nerve signals may also contribute to maintaining the stability of the pro-repair phenotype and tissue reconstruction (99). Therefore, the sympathetic nervous system should not be simply categorized as promoting or inhibiting bone repair, but should be understood in conjunction with the phase, local concentration, receptor expression, and microenvironmental state.

Overall, sensory neuropeptides and sympathetic nerve signals jointly regulate the timing and direction of the macrophage functional shift. If neural signal input is insufficient, imbalanced, or chronically dysregulated, macrophages may remain in a pro-inflammatory clearance state for an extended period, compromising inflammatory switching and subsequent bone regeneration; conversely, appropriate neural regulation may contribute to the establishment of a pro-repair microenvironment (100–102) (**Table 1**).

### 5.2 Constructing an immune microenvironment conducive to osteogenesis

The significance of the macrophage functional shift extends beyond the attenuation of inflammation; it is also crucial for reshaping the local osteogenic microenvironment through the secretion of pro-repair factors (109). M2-like/pro-repair macrophages can release molecules such as TGF-β, BMP-2, VEGF, and IL-10, linking the resolution of inflammation with angiogenesis, cell recruitment, and osteogenic activities (110, 111). Among these, TGF-β and BMP-2 are primarily involved in the recruitment of bone mesenchymal stem cells (BMSCs), their osteogenic differentiation, and matrix deposition; VEGF, by promoting the formation of new blood vessels, provides oxygen, nutrients, and conduits for cell migration for the callus tissue (112). Therefore, the microenvironment constructed by pro-repair macrophages is not a single “anti-inflammatory environment” but a regenerative microenvironment that simultaneously supports vascularization and osteogenic differentiation.

Neural signals can further modulate this network of pro-repair factors. On one hand, neuropeptides derived from sensory nerves may enhance local repair signals such as IL-10, TGF-β, and VEGF by regulating the macrophage state (113); on the other hand, some neural signals can also directly act on BMSCs and vascular endothelial cells, translating changes in the immune microenvironment into osteogenic and vascularization effects (114). This process suggests that neural regulation and immune regulation are not parallel events but are coupled within the local repair microenvironment. Furthermore, the pro-repair microenvironment also requires maintaining a balance between osteogenesis and osteoclastogenesis. If the pro-inflammatory state persists, a dysregulation of the RANKL/OPG ratio, enhanced osteoclast activity, and suppressed osteogenesis may collectively hinder bone regeneration (115, 116). Therefore, optimal neuro-immune regulation is not merely focused on enhancing one particular macrophage phenotype, but on coordinating the resolution of inflammation, vascularization, osteogenic differentiation, and bone remodeling within the appropriate spatiotemporal context.

### 5.3 Coordinating the tripartite dialogue among nerves, immunity, and blood vessels

The successful reshaping of the bone regeneration microenvironment is not a mere summation of the independent functions of the neural, immune, and vascular modules, but a positive feedback loop established via their continuous dialogue (117). In other words, the neuro-immune-vascular triad does not function as three parallel modules; rather, it governs the efficiency and quality of bone regeneration through a positive feedback loop. Within this loop, neural signals can first directly promote angiogenesis. Neuropeptides released by sensory nerves, such as CGRP and SP, can act on vascular endothelial cells, promoting their proliferation, migration, and tube formation, thereby accelerating angiogenesis in the injured area (118, 119). Furthermore, new blood vessels are not merely conduits for material supply; their endothelial cells can also secrete various neurotrophic factors and repair-related molecules, providing support for axonal extension and local neural remodeling (120). Consequently, a characteristic bidirectional promotive relationship emerges between nerves and blood vessels.

The immune system, particularly M2-like macrophages, serves as a key mediator for amplifying this loop (121). Polarized macrophages can secrete pro-angiogenic and pro-repair factors such as VEGF and TGF-β, thereby further strengthening the vascular network while also creating a more stable microenvironment for effective neural signal transmission and cell migration (122, 123). Conversely, a well-established vascular system improves the local transport efficiency of neuropeptides, inflammatory mediators, and nutritional signals, allowing the neuro-immune dialogue to continue at a higher level (124). The net result is a cycle wherein nerves promote vasculature, vasculature supports innervation, and the immune system amplifies and stabilizes this interaction, collectively driving the transition of bone regeneration from an inflammatory state to an efficient repair state. Therefore, the essence of the tripartite dialogue among nerves, immunity, and blood vessels lies not in the strongest effect of any single factor, but in their formation of a mutually reinforcing regenerative network. This network determines both whether local inflammation can be effectively resolved and whether osteogenesis and vascularization can proceed synchronously. For post-traumatic bone regeneration, truly high-quality repair is not merely the formation of new bone, but the reconstruction of structure and microenvironment based on this tripartite coupling.

### 5.4 Direct association between axis dysregulation and clinical complications

When the regulation of the neuro-immune axis is imbalanced, clinical outcomes may manifest as delayed bone healing, non-union, chronic pain, or poor functional recovery. The core issue is not a single molecular abnormality, but the failure of the local regenerative program to effectively transition from a persistent inflammatory state to a tissue reconstruction state.

At the level of bone repair, reduced local innervation, decreased levels of key neuropeptides, or impaired macrophage functional shift can all cause the repair microenvironment to remain in a pro-inflammatory and hypo-repair state for a prolonged period (64). For example, insufficient CGRP signaling may simultaneously affect local vascular responses, macrophage functional state, and osteoblast activity, thereby increasing the risk of insufficient blood supply, suppressed osteogenesis, and poor callus formation. Such changes can render the bone regeneration process persistently inefficient, increasing the likelihood of delayed union or non-union (125, 126). Consequently, osteoblast differentiation is inhibited, vascular restoration is compromised, and the establishment of a conducive bone regeneration microenvironment is hindered, collectively elevating the risk of delayed union and non-union.

**Figure 4:**
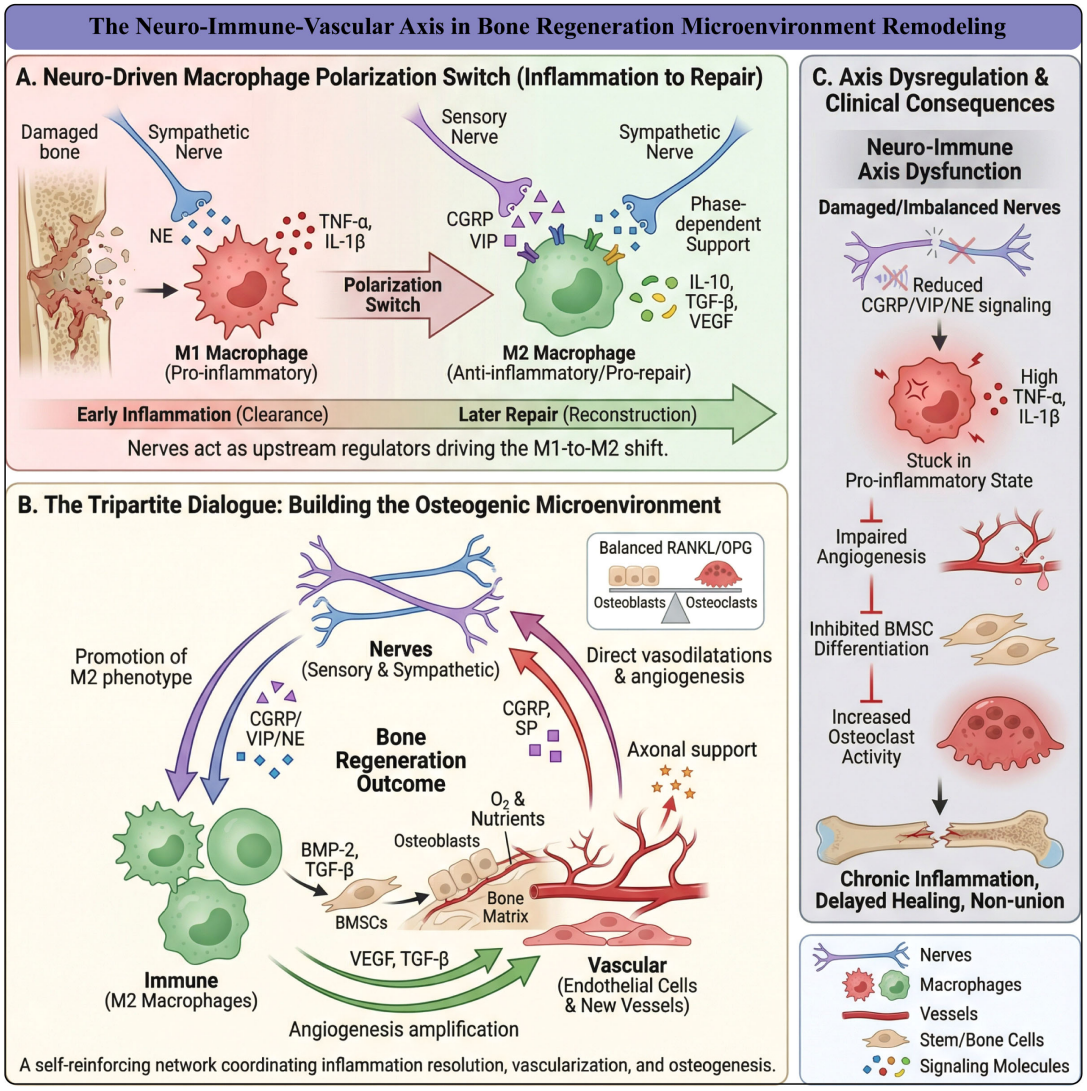
The neuro-immune-vascular axis in the reshaping of the bone regeneration microenvironment. **(A)** After injury, sympathetic and sensory nerves regulate the conversion of macrophages from a pro-inflammatory M1-like state to a pro-repair M2-like state via signals such as NE, CGRP, and VIP, driving the transition from inflammatory clearance to tissue reconstruction. **(B)** A mutually reinforcing multicellular crosstalk emerges between nerves, M2-type macrophages, vascular endothelial cells, and BMSCs: neural signals promote M2 polarization and vasodilation; M2 Macrophages and blood vessels jointly support osteogenic differentiation, angiogenesis, and the RANKL/OPG balance, thereby optimizing the osteogenic microenvironment and promoting bone repair. **(C)** When the neuro-immune axis is imbalanced, CGRP/VIP/NE signaling is weakened, macrophages are retained in a pro-inflammatory state, leading to impaired angiogenesis, inhibited BMSC differentiation, and enhanced osteoclast activity, ultimately resulting in chronic inflammation, delayed union, and non-union.

Besides affecting bone healing, dysregulation of the neuro-immune axis can also drive persistent neurogenic inflammation and immune activation (127, 128). If sensory nerves are persistently abnormally excited, or if local pro-inflammatory macrophages persist for a long time, inflammatory signals will be continuously maintained and amplified, creating a microenvironment unfavorable for tissue reconstruction (129). The core of this pathological state is not simply “more severe inflammation,” but the failure of inflammation to switch to a repair program. The direct consequence of this is that the bone regeneration process becomes arrested in an inefficient or even imbalanced state for an extended period, with osteogenesis, vascularization, and neural remodeling concurrently inhibited (130, 131). Therefore, from a mechanistic perspective, the key consequence of neuro-immune axis dysregulation is not a single specific complication, but the local regenerative program remaining locked in a maladaptive “pro-inflammation–hypo-repair” cycle (132). Consequently, restoring the normal temporal regulation of the neuro-immune axis is not only necessary for promoting bone healing but also a crucial prerequisite for avoiding a cascade of subsequent repair obstacles (**Figure 4**).

## 6 Post-traumatic neuro-immune axis in functional recovery: from bone healing to pain and motor recovery

### 6.1 Expansion of bone regeneration evaluation systems: equal emphasis on structural repair and functional recovery

The success of bone regeneration lies not only in the restoration of bone tissue structure but also in the comprehensive rehabilitation of limb function. Traditional evaluation systems for bone regeneration primarily focus on structural repair indicators, such as assessing callus volume, bone mineral density, and trabecular microstructure through imaging methods (e.g., X-ray, Micro-CT) (133), as well as evaluating the biomechanical properties of new bone through biomechanical testing (e.g., torsional strength, three-point bending test) (134). These indicators are crucial for assessing the biological process of bone healing. For instance, studies have confirmed that the Wnt signaling pathway-related marker Lgr6 is essential for maintaining bone volume and efficient bone regeneration (135), while bone morphogenetic protein-2 (BMP-2) significantly promotes osteoblast differentiation and osteogenesis at bone defects (136). However, these traditional indicators fail to comprehensively reflect the patient’s functional recovery status, such as pain relief, weight-bearing capacity, joint range of motion, muscle strength, and motor coordination. Therefore, the evaluation of bone regeneration should be expanded from simple structural healing to functional endpoints including pain, weight-bearing, joint mobility, and motor recovery.

The neuro-immune axis warrants special attention precisely because it lies at the intersection of structural repair and functional recovery. At the structural repair level, the neuro-immune axis directly influences the osteogenic process and bone healing quality by regulating inflammatory switching, macrophage polarization, and local microenvironment stability; at the functional recovery level, it profoundly affects post-traumatic activity recovery and rehabilitation processes by modulating pain perception, neuroplasticity, and local reinnervation (137). For example, impaired lymphatic drainage can lead to restricted clearance of DAMPs and persistent inflammation, thereby weakening bone healing; conversely, more orderly inflammation resolution and increased M2-like macrophages are beneficial for osteoblast survival and mesenchymal stem cell proliferation (138). Therefore, evaluating repair outcomes solely based on imaging or biomechanical indicators often fails to fully reflect the true clinical benefits after trauma. In the future, a more comprehensive evaluation framework should combine imaging and biomechanical indicators with assessments of pain, weight-bearing, functional capacity, and motor function to more comprehensively capture the true outcomes of bone regeneration.

### 6.2 Neuro-immune axis and post-traumatic pain sensitization and dysfunction

Post-traumatic pain sensitization is one of the most prominent functional consequences of neuro-immune axis imbalance, with its core mechanism summarized as a continuous amplification of peripheral sensitization and central sensitization (139). In the early stage of injury, inflammatory mediators and DAMPs released from damaged tissue directly activate and sensitize peripheral nociceptive sensory nerve endings. These sensory neurons, particularly nociceptors expressing channels such as TRPV4, release neuropeptides like SP and CGRP upon activation (140). These neuropeptides not only directly lower the excitation threshold of peripheral sensory neurons, leading to amplified pain signals, but also act on the local immune microenvironment, recruiting and activating immune cells such as macrophages and mast cells, inducing the release of pronociceptive inflammatory factors like IL-1β and TNF-α (141). These pro-inflammatory mediators, in turn, further sensitize sensory nerve endings, forming a positive feedback loop of “neural activation—immune amplification—resensitization,” thereby driving the persistence of peripheral sensitization.

As abnormal nociceptive input is continuously transmitted through the dorsal root ganglia to the spinal dorsal horn, pain processing enters the central sensitization stage (142). At this point, microglia and astrocytes at the spinal level are persistently activated, enhancing the excitability of spinal dorsal horn neurons and inducing synaptic plasticity by releasing mediators such as lipocalin-2, IL-1β, and TNF-α, thereby amplifying and maintaining pain signal transmission (143, 144). Therefore, post-traumatic pain is not merely a sensory abnormality but the result of continuous amplification of neuro-immune interactions. Its clinical significance lies in the fact that the persistence of pain often does not entirely depend on the degree of structural damage at the fracture site but rather reflects whether the neuro-immune axis remains in an abnormally activated state. More importantly, pain sensitization can, in turn, impair bone regeneration and rehabilitation itself. Persistent pain can significantly reduce the patient’s willingness to bear weight and decrease their activity level, inducing protective disuse, leading to further muscle atrophy, joint stiffness, and abnormal movement patterns in the affected limb (145). In other words, post-traumatic pain is not only a consequence of poor repair but also a driving factor for repair impairment (146). This “pain-inflammation-disuse” chain explains why some patients, even with basic radiological bone union, still have unsatisfactory overall functional outcomes.

### 6.3 Negative impact of persistent inflammation on neural recovery and motor function

In the process of hindered post-traumatic functional recovery, persistent inflammation is a crucial intermediate link connecting structural repair and dysfunction (147). If the local inflammatory response cannot promptly switch from a pro-inflammatory state to a pro-repair state, nerve regeneration and reinnervation become significantly restricted (148). Studies have shown that a sustained pro-inflammatory microenvironment can upregulate the expression of inhibitory molecules. For instance, expression of the axon guidance molecule Semaphorin 3A is significantly upregulated following peripheral nerve injury; this upregulation correlates with inflammatory factor levels and directly inhibits axon extension (149). In models such as spinal cord injury, persistent neuroinflammation is also closely associated with glial scar formation, which impedes axon crossing of the injury zone through both physical and chemical barriers (150). Furthermore, persistent inflammation can exacerbate Wallerian degeneration and inhibit the repair response of Schwann cells, thereby delaying nerve debris clearance and the preparation of the regenerative microenvironment (151). These changes collectively suggest that if the sustained pro-inflammatory state cannot be resolved in a timely manner, neural recovery itself will be significantly delayed.

**Figure 5:**
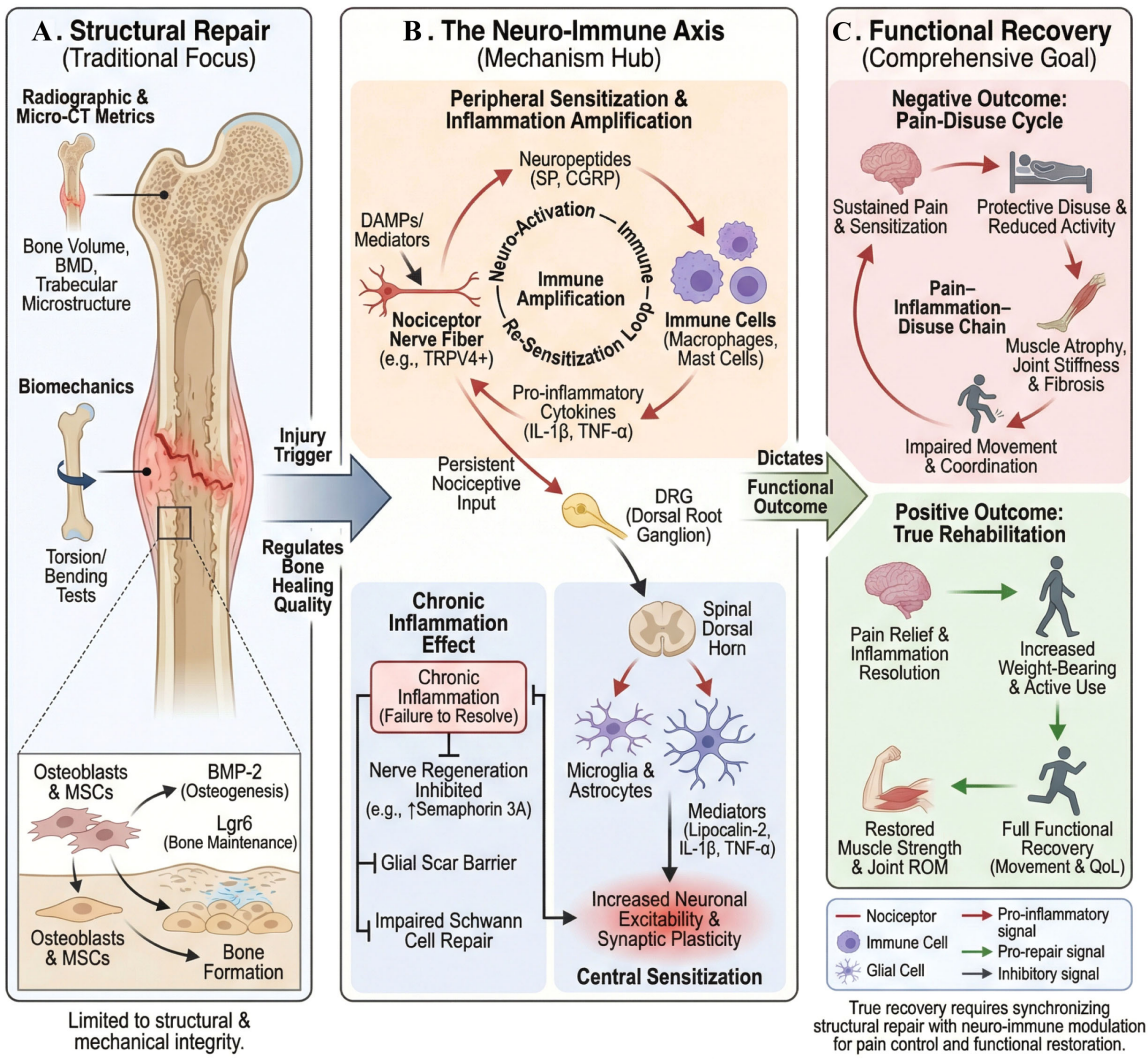
How the neuro-immune axis links the structural outcomes of bone repair with pain control and motor recovery. **(A)** Traditional bone healing assessment mainly focuses on imaging, bone density, trabecular structure, and biomechanical indicators (Biomechanical analysis) to judge the degree of bony structural repair. **(B)** After injury, DAMPs and inflammatory mediators activate nociceptive nerves and immune cells, forming a peripheral sensitization-inflammation amplification loop mediated by SP, CGRP, IL-1β, and TNF-α, while simultaneously driving central sensitization through dorsal root ganglia, spinal dorsal horn, and glial cell responses; persistent inflammation can also inhibit nerve regeneration and hinder Schwann cell repair. **(C)** If the neuro-immune imbalance persists, a vicious cycle of pain-inflammation-disuse will form, leading to muscle atrophy, joint stiffness, and dysfunction; conversely, if inflammation resolution and pain relief are achieved, weight-bearing, muscle strength, and joint range of motion can be restored, ultimately promoting true functional rehabilitation.

This persistent inflammation does not stop at the neural structural level but further manifests as more pronounced motor dysfunction (152). The sustained pro-inflammatory state and impaired neural recovery not only exacerbate pain sensitization but also weaken sensory input and motor control, ultimately leading to reduced use of the affected limb, decreased muscle strength, limited joint mobility, and poorer coordination (153). In other words, persistent inflammation, pain perpetuation, and functional decline are not independent issues but a continuous process of reciprocal amplification. Clinical and experimental studies both indicate that the more pronounced the pro-inflammatory environment, the worse the neural functional recovery tends to be; conversely, pro-resolving interventions can not only alleviate pain but also improve nerve regeneration and motor recovery (154). Therefore, post-traumatic functional recovery impairment is not simply a direct result of “incomplete bone healing” but rather the outcome of a self-reinforcing closed loop of “persistent inflammation-impaired neural recovery-pain maintenance-increased disuse” caused by neuro-immune axis imbalance. From this perspective, the endpoint of trauma repair should not be defined solely by bony union but should include pain control, neural recovery, and motor function reconstruction. Even if the bone has achieved biological union, without effective neural innervation, effective pain management, and sufficient functional integration, the patient is unlikely to achieve true rehabilitation (155). Therefore, in post-traumatic bone regeneration research and clinical intervention, synchronously regulating the neuro-immune axis to achieve the unity of structural repair and functional recovery should be regarded as a higher-level therapeutic goal (**Figure 5**).

## 7 Intervention strategies for promoting bone regeneration by targeting the neuro-immune axis

**Figure 6:**
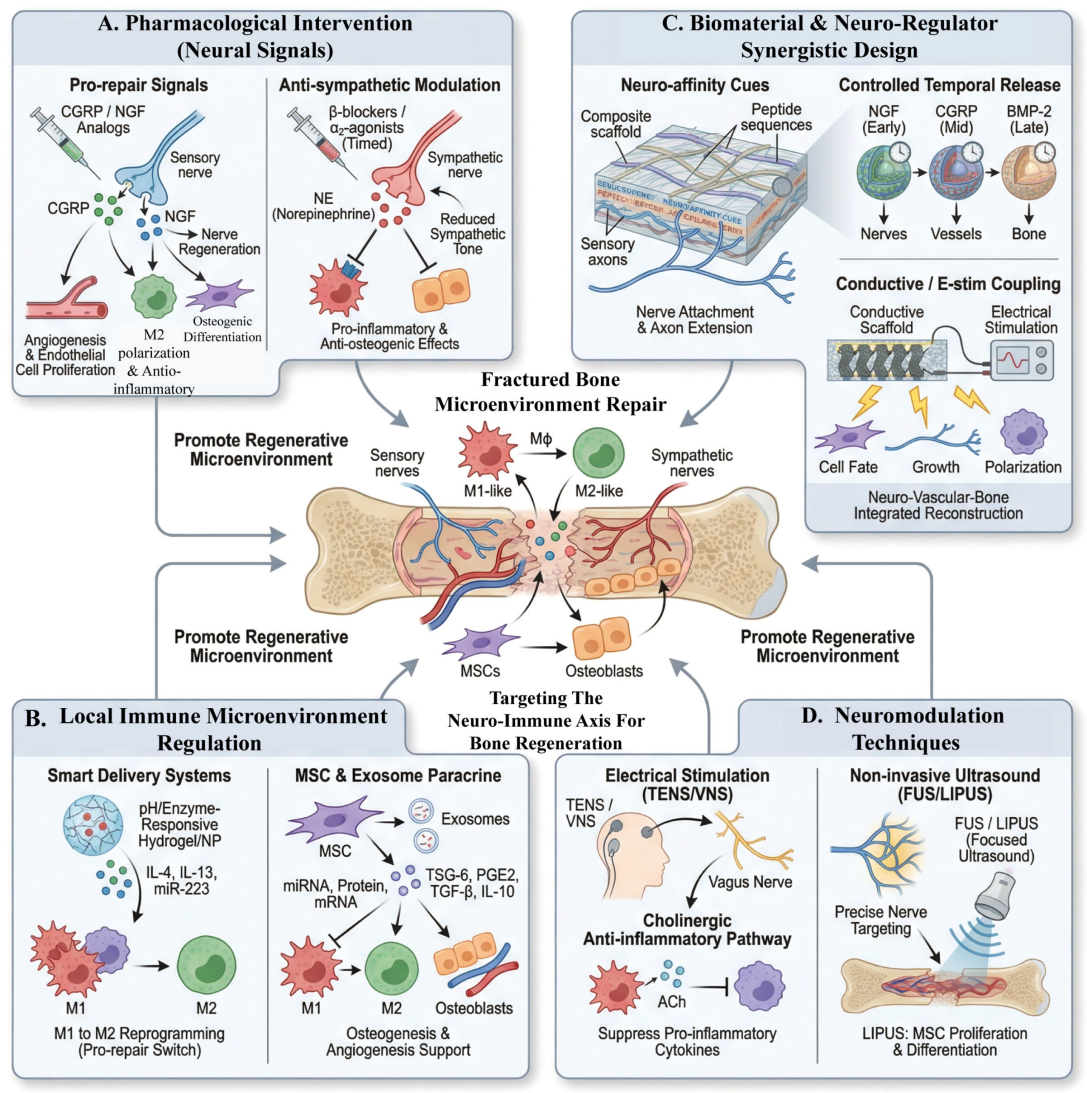
Comprehensive intervention strategies for promoting bone regeneration by targeting the neuro-immune axis. **(A)** Pharmacological intervention promotes angiogenesis, M2 macrophage polarization, and osteogenic differentiation by supplementing pro-repair neural signals (e.g., CGRP/NGF) or temporarily inhibiting sympathetic activity. **(B)** Shows that local immune microenvironment modulation can utilize intelligent delivery systems and MSC/exosome paracrine effects to drive macrophage transformation from M1 to M2 and support osteogenesis and angiogenesis. **(C)** Shows the synergistic design of biomaterials and neuroregulatory factors, promoting axon extension and neuro-vascular-bone integrated reconstruction through neuro-affinity scaffolds, time-controlled release, and conductive/electrical stimulation coupling. **(D)** Shows that neuromodulation techniques such as electrical stimulation (TENS/VNS) and focused ultrasound/LIPUS can inhibit inflammation and enhance MSC proliferation and differentiation through the cholinergic anti-inflammatory pathway and precise neural targeting. Overall, these strategies collectively promote bone repair by optimizing the fracture microenvironment and reconstructing the neuro-immune-vascular-bone coupling network.

Based on the above mechanisms, targeting the neuro-immune axis has become an important therapeutic strategy for promoting post-traumatic bone regeneration. Compared with traditional strategies focusing primarily on osteogenesis or material replacement, these approaches emphasize the synergistic improvement of bone healing and functional recovery through the regulation of neural signals, inflammatory switching, and local microenvironment remodeling. However, their clinical translation still requires comprehensive consideration of dose range, intervention window, delivery method, safety, off-target effects, patient heterogeneity, and operational feasibility. Current research mainly involves pharmacological intervention, local immune microenvironment modulation, synergistic design of biomaterials, and neuromodulation techniques, but the evidence base and translational maturity of different strategies vary. Based on the source of evidence and clinical proximity, this article stratifies relevant strategies into four levels: Level I includes strategies with existing clinical trials for bone repair or relatively clear clinical application foundations; Level II includes strategies with early clinical studies or clinical data from adjacent indications but insufficient evidence for post-traumatic bone regeneration; Level III includes preclinical strategies mainly based on animal experiments; Level IV includes strategies primarily based on *in vitro* experiments, mechanistic inference, or extrapolation from other disease fields. The following sections compare and evaluate different intervention directions based on evidence level, advantages, limitations, and clinical data (**Figure 6**).

### 7.1 Pharmacological intervention based on neural signaling molecules

Pharmacological intervention based on neural signaling molecules is one of the important strategies for regulating the post-traumatic neuro-immune axis and promoting bone regeneration. Neuropeptides and neurotransmitters released by nerve endings directly participate in the regulation of the bone repair microenvironment, among which CGRP and NGF are representative pro-repair signaling molecules (156). Current studies indicate that CGRP can promote vascular endothelial cell proliferation and migration by activating its receptor, accelerate angiogenesis in the fracture area, and help guide macrophages towards an anti-inflammatory, pro-repair phenotype, thereby alleviating early excessive inflammation and creating favorable conditions for subsequent osteogenesis (157). NGF promotes both sensory nerve regeneration and the differentiation of osteogenic precursor cells, enhancing the regenerative potential of the bone defect site by upregulating related signaling pathways (158). Therefore, local supplementation of CGRP analogs or NGF holds potential to synergistically enhance angiogenesis, immune regulation, and osteogenic differentiation by mimicking physiological neural signals (159). From a comparative perspective, the main advantage of CGRP/NGF-based strategies lies in their clear targets, simultaneously connecting nerve reinnervation, angiogenesis, and osteogenesis; their limitations include unresolved issues with half-life, local dose control, delivery stability, and potential risk of abnormal neural sensitization (160). Currently, these strategies are mainly at the Level III or IV evidence stage, primarily based on animal experiments and mechanistic studies, lacking high-quality clinical trial data for post-traumatic bone regeneration.

A key translational issue for this class of drugs is determining the therapeutic window between the locally effective dose and the safe dose. If the NGF/CGRP dose is too low, it may be insufficient to rebuild neuro-vascular-bone repair signals; if the dose is too high, the release range is too broad, or the duration is too long, it may cause pain sensitization, abnormal nerve sprouting, abnormal vascular reactions, or neurogenic inflammation (161). It should be noted that long-term or excessive supplementation of NGF/CGRP carries significant safety concerns. NGF promotes sensory nerve growth and neural sensitization; improper control of dose, timing, or local release range may increase the risk of pain sensitization, abnormal nerve sprouting, or neurogenic inflammation (162). Although CGRP has pro-angiogenic and pro-repair effects, long-term or excessive elevation may also affect vascular tone, local inflammatory responses, and pain transmission (163, 164). Therefore, neuropeptide interventions are more suitable for local, short-term, and controlled release strategies, rather than simple long-term systemic supplementation. Future research should simultaneously evaluate pro-repair effects and potential adverse reactions, including safety endpoints such as pain sensitization, abnormal nerve reinnervation, excessive vascular reactions, and chronic neurogenic inflammation. Additionally, priority should be given to clarifying the administration window, local delivery method, dose-response relationship, and adverse reaction threshold.

Besides directly supplementing pro-repair signals, pharmacological regulation of the autonomic nervous system, particularly sympathetic nerve activity, represents a promising avenue worthy of investigation (165). Post-traumatic sympathetic nerves are often overactivated, and the release of catecholamine transmitters like norepinephrine may mediate pro-inflammatory and anti-osteogenic effects through adrenergic receptors, thus being detrimental to fracture healing. Based on this, applying selective β-adrenergic receptor blockers or α2-receptor agonists within a specific time window is considered to optimize the bone regeneration microenvironment by reducing excessive sympathetic tone, alleviating inflammatory factor release, and lifting the inhibition on osteoblasts (166, 167). However, the role of sympathetic nerves in bone repair has significant phase-dependent and bidirectional characteristics; excessive inhibition may also interfere with physiological repair programs (168, 169). Compared with neuropeptide supplementation, the advantage of autonomic nervous system pharmacological regulation lies in the fact that some drugs already have a clinical use basis, with relatively more pharmacokinetic and safety data; its limitation is the broad range of action, making it difficult to achieve specific regulation localized to the bone injury, and different repair stages may require different regulation intensities.

Autonomic nervous system pharmacological regulation is also subject to distinct safety constraints. β-blockers or α2-receptor agonists may affect cardiovascular responses, local blood perfusion, and systemic stress adaptation; excessive inhibition of sympathetic activity may also interfere with the necessary inflammatory initiation, vascular response, and tissue clearance processes in the early stages of trauma (170). Furthermore, autonomic drugs may produce systemic off-target effects, including changes in heart rate and blood pressure, decreased local perfusion, suppressed stress response, and reduced tolerance in patients with multi-system injuries (171). Therefore, such strategies necessitate well-defined indications, precise administration windows, appropriate dose ranges, and a thorough assessment of patient baseline status, with particular attention to potential risks in elderly patients, patients with cardiovascular disease, and those with combined multi-system injuries. Current evidence is derived primarily from basic research, animal models, or indirect clinical observations, as there is a paucity of prospective clinical trials utilizing bone regeneration outcomes as the primary endpoint. Thus, this direction can be classified as Level II-III evidence, with translational potential, but clinical application scenarios and safety boundaries still need further clarification.

### 7.2 Strategies for regulating the local immune microenvironment

Interventions targeting the local immune microenvironment around the neuro-immune axis essentially involve reprogramming the post-traumatic inflammatory switching process, shifting the injury site from a sustained pro-inflammatory state to a pro-repair state conducive to osteogenesis and vascularization (172). Current research mainly proceeds along two paths. One relies on intelligent delivery systems, using pH- or enzyme-responsive hydrogels, nanoparticles, and other multifunctional carriers to locally deliver M2 polarization-inducing factors such as IL-4 and IL-13, or immune-regulating molecules like miR-223, to actively guide macrophage transformation from M1 to M2 (173, 174). In recent years, injectable hydrogels with dual antibacterial and immunomodulatory functions have also been used for infected bone defect repair, providing new material approaches for open trauma, infected bone defects, or complex bone regeneration scenarios by regulating the antibacterial/immune microenvironment at the bone-hydrogel interface (175). Additionally, the material’s own surface topography, pore size, stiffness, degradation rate, charge, and hydrophilicity can influence macrophage adhesion, activation, and functional phenotype. Therefore, immunomodulatory material design should not only focus on delivery factors but also consider the direct shaping effect of material characteristics on the immune microenvironment (176). The advantage of these local delivery strategies lies in strong targeting, combinability with material release systems, and potential for staged immune regulation; their limitations include unresolved issues with delivery efficiency, release kinetics, batch consistency, undesired immune skewing, and long-term safety. In particular, the key to immune microenvironment regulation is not simply enhancing the pro-repair response, but precisely orchestrating the transition between inflammatory clearance and the onset of repair (24). Prematurely inhibiting the pro-inflammatory response in cases of infection, open trauma, or incomplete debridement may weaken antibacterial defense and debris clearance; maintaining a pro-repair shift for a long time may increase the risk of fibrosis, abnormal vascularization, or abnormal tissue remodeling (177). Therefore, such strategies should be applied in a stratified manner based on infection status, soft tissue injury degree, host immune status, and bone defect type, currently remaining closer to Level III preclinical evidence.

Another strategy leverages cell paracrine effects to regulate the local inflammatory microenvironment, with mesenchymal stem cells (MSCs) and their derived exosomes representing the most prominent examples (178). The value of MSCs lies not only in their differentiation potential but also in their ability to inhibit excessive pro-inflammatory responses by releasing factors such as TSG-6, PGE2, TGF-β, and IL-10, promote the formation of a reparative immune state, and support osteogenesis and angiogenesis (179, 180). Building on this, MSC-derived exosomes, as key paracrine carriers, can deliver active molecules such as proteins, mRNA, and miRNA, playing roles in immune regulation, osteogenesis promotion, and potential neurotrophic support (181, 182).

Compared with single cytokine delivery, MSC/exosome strategies can simultaneously affect inflammation, vascularization, osteogenesis, and neurotrophic support, but also face issues such as cell source differences, non-uniform preparation processes, difficulty in defining active components, limited *in vivo* homing efficiency, and uncertain long-term safety (183). Additionally, potential risks like immunogenicity, batch heterogeneity, excessive angiogenesis, ectopic osteogenesis, and abnormal tissue remodeling may be more prominent in patients with diabetes, osteoporosis, infected bone defects, or abnormal immune function (184, 185). Therefore, future research needs to incorporate patient heterogeneity into efficacy and safety assessments. Overall, MSCs have some early clinical exploration in the fields of bone defects, non-union, or tissue repair, and can be considered Level II-III evidence; while direct clinical evidence for MSC exosomes in post-traumatic bone regeneration, especially neuro-immune axis regulation, remains scarce, placing them closer to Level III evidence.

### 7.3 Synergistic design of biomaterials and neuroregulatory factors

Rather than understanding this field as continuously adding “new materials,” it is better summarized as synergistic design around several core principles. First, ideal bone regeneration materials need to possess neuro-affinity, meaning they can provide a suitable microenvironment for nerve fiber ingrowth, neurotrophic factor action, and neurovascular reconstruction (186). Particularly, biomimetic periosteal materials, due to their ability to mimic the structural barrier, vascularization support, and osteogenic regulatory functions of the natural periosteum, have become an important direction for large segmental bone defect and complex bone repair material design (187). Consequently, materials often enhance their support for nerve regeneration by mimicking natural extracellular matrix components or introducing structural features conducive to nerve attachment and axon extension (188, 189). Meanwhile, the material’s pore structure, surface roughness, mechanical stiffness, and degradation behavior not only affect cell adhesion and osteogenic differentiation but also alter macrophage M1/M2 phenotypes and the inflammatory microenvironment, thus should be incorporated into the integrated neuro-immune-bone material design framework. Second, such materials typically emphasize controlled release.

Sequentially delivering neuro/osteogenic factors like NGF, CGRP, or BMP-2 can support nerve reinnervation, angiogenesis, and osteogenic differentiation at different repair stages, avoiding burst release and phase mismatch issues associated with single administration (160). In terms of evidence level, some traditional bone repair materials and osteogenic factors like BMP-2 have certain clinical application or clinical research foundations and can be classified as Level I-II evidence; however, the design of synergistic neuro-immune-bone delivery of NGF, CGRP, immune regulatory factors, and materials is still mainly in the animal experiment and material validation stage, overall belonging to Level III evidence. The advantages of this approach include enabling local delivery, spatial localization, and staged release; limitations include system complexity, incompletely understood regulatory mechanisms, high production consistency requirements, and potential difficulties in dose matching and safety assessment for multi-factor combined delivery (190, 191). For composite biomaterials, safety depends not only on the individual factor but also on the combined effects of material degradation products, release kinetics, multi-factor dose matching, and local tissue response (192). If factors like NGF, CGRP, and BMP-2 are released too quickly, at excessively high local concentrations, or for too long, they may cause abnormal vascularization, pain sensitization, ectopic bone formation, or inflammatory reactions (193). Therefore, future material design should not only evaluate pro-repair effects but also simultaneously clarify the effective dose window, release duration, and adverse reaction boundaries.

Third, increasing research in recent years has focused on the design principle of conductive/electrical stimulation coupling. Since nerves and bones are both electroactive tissues, conductive materials combined with exogenous electrical stimulation are expected to simulate the local physiological electrical signal environment, thereby regulating stem cell fate, nerve growth, and macrophage polarization (194). Recent studies further show that piezoelectric stimulation can promote alveolar bone defect repair by reshaping macrophage metabolic status, suggesting that electroactive materials may not only affect osteoblast behavior but also participate in bone regeneration regulation through immune metabolic pathways (195). The focus of this design is not on the “novelty” of the material itself, but on whether it can promote the synergistic occurrence of nerve repair and bone regeneration by transmitting or amplifying bioelectrical signals (196). Finally, the ultimate objective is to achieve integrated neuro-vascular-bone reconstruction. Specifically, biomaterial design should not only prioritize new bone formation but also consider the restoration of nerve innervation, establishment of vascular networks, and optimization of the immune microenvironment to support higher-quality structural repair and functional recovery (157). Recent advances in craniomaxillofacial tissue engineering also suggest that future bone regeneration materials need to simultaneously address issues of scaffold structure, vascularization, immune regulation, personalized manufacturing, quality control, and clinical scalability, which are highly consistent with the translational challenges of integrated neuro-immune-bone material design (197).

The advantage of conductive materials and electrical stimulation coupling strategies lies in their ability to simulate the electrophysiological microenvironment of bone repair and modulate cell behavior, nerve growth, and immune regulation; however, their stimulation parameters, conductive stability, tissue interface response, and long-term implantation safety still require further elucidation (198). If material stability is insufficient, or electrical stimulation intensity, frequency, and duration are improperly controlled, it may lead to local tissue irritation, abnormal nerve excitation, inflammatory reactions, or adverse material interface responses (199). Traditional electrical stimulation for delayed fracture healing or non-union has an established foundation in clinical practice and can be considered Level I-II evidence; however, composite strategies combining conductive scaffolds with neuro-immune regulatory factors are primarily classified as Level III evidence and thus remain distant from widespread clinical adoption.

### 7.4 Application of neuromodulation techniques

Besides drugs and biomaterials, neuromodulation techniques provide another non-pharmacological pathway for intervening in the post-traumatic neuro-immune axis (200). Transcutaneous electrical nerve stimulation (TENS) and vagus nerve stimulation (VNS) are two relatively well-studied methods (201). Their theoretical basis lies in regulating the activity of specific neural pathways to influence the intensity and timing of the inflammatory response, thereby creating a more favorable immune microenvironment for bone regeneration. For example, the cholinergic anti-inflammatory pathway is considered an important bridge connecting the nervous system and immune regulation; TENS or VNS may enhance acetylcholine-related signals, inhibit excessive release of pro-inflammatory factors from immune cells such as macrophages, and promote the transition of inflammation from an exacerbated state to a controlled repair state (202, 203). From a translational perspective, the advantages of TENS include non-invasiveness, ease of operation, relatively high safety, and extensive clinical experience in pain management and rehabilitation; however, its direct promoting effect on bone regeneration and neuro-immune mechanisms still lack sufficient validation (204). VNS has a relatively clear basis in the cholinergic anti-inflammatory pathway, but its invasiveness, device dependence, optimization of stimulation parameters, and selection of suitable populations constrain its direct application in bone regeneration (205). Additionally, VNS may affect heart rate, blood pressure, and autonomic function, requiring careful assessment, especially in elderly patients, those with cardiovascular disease, or multiple trauma patients (206). Overall, while TENS and VNS represent Level II evidence, they cannot yet be regarded as established treatment protocols for bone regeneration.

Non-invasive neuromodulation techniques such as focused ultrasound represent a more cutting-edge direction. Compared with traditional electrical stimulation, focused ultrasound offers higher spatial resolution and tissue penetration, theoretically enabling non-invasive precise regulation of specific nerve bundles or local neural networks (207). Low-intensity pulsed ultrasound (LIPUS) has shown potential in promoting mesenchymal stem cell proliferation, migration, and osteogenic differentiation, also providing inspiration for developing more precise nerve-targeted ultrasound regulation (208). It is important to distinguish that while LIPUS has an established foundation in fracture healing, its efficacy varies across studies and indications, placing it at Level I-II evidence. In contrast, focused ultrasound, as a precise tool for regulating the neuro-immune axis in bone regeneration, currently remains primarily at the Level III-IV evidence stage (209).

Although neuromodulation techniques offer advantages such as being non-pharmacological, non-invasive, or minimally invasive, they also have significant parameter-dependent risks. Excessive or inappropriate electrical/ultrasound stimulation may cause increased pain, abnormal nerve excitation, local tissue irritation or damage, and even interfere with necessary early inflammatory and vascular responses (210). For focused ultrasound and LIPUS, acoustic intensity, frequency, pulse mode, depth of action, and number of treatments can all affect their biological effects; improper parameter selection may lead to local thermal effects, excessive mechanical stress, or non-target tissue reactions. For such techniques, “dose” is mainly reflected in stimulation intensity, frequency, duration, depth of action, and treatment schedule (211). Differences in pain sensitivity, degree of nerve injury, internal fixation method, soft tissue status, and comorbidities among patients may lead to different effects from the same parameters. Therefore, before clinical translation, it is necessary to clarify standardized parameters, contraindications, monitoring indicators, and the method of integration with rehabilitation treatment (**Table 2**).

**Table 2:** Comparative evaluation of intervention strategies targeting the neuro-immune axis to promote bone regeneration after trauma.

Intervention category	Representative strategies	Main mechanisms of action	Level of evidence	Main advantages	Main limitations & safety issues	Clinical data status	Reference
Pharmacological intervention with neural signaling molecules	Local delivery of CGRP, NGF.	Mimics pro-repair signals from nerve endings, promoting reinnervation, angiogenesis, immune regulation, and osteogenic differentiation.	Level III–IV	Clear targets; can simultaneously connect nerve, vascular, immune, and bone repair processes.	Short half-life; difficult to control local dose and release timing; excessive or prolonged release may increase the risk of pain sensitization, aberrant nerve sprouting, abnormal vascular response, or neurogenic inflammation.	Currently mainly animal experiments and mechanistic studies; lacks high-quality clinical trials related to post-traumatic bone regeneration.	(157–164)
Pharmacological regulation of autonomic nerves	Beta-blockers, alpha-2 receptor agonists	Reduces excessive sympathetic tone, attenuates release of inflammatory cytokines and anti-osteogenic effects mediated by sympathetic signals.	Level II–III	Some drugs have a clinical use foundation; relatively more pharmacokinetic and safety data available.	Broad range of action, difficult to achieve specific local regulation in bone injury; may affect heart rate, blood pressure, local perfusion, and systemic stress adaptation; excessive inhibition of sympathetic activity may interfere with early inflammation initiation and tissue clearance.	Has clinical application foundation in adjacent fields, but lacks prospective clinical trials with bone regeneration as the primary endpoint.	(166–171)
Regulation of local immune microenvironment	pH/enzyme-responsive hydrogels, nanoparticles delivering IL-4, IL-13, miR-223, etc.	Induces macrophage phenotype switch from M1-like pro-inflammatory to M2-like pro-repair, promoting inflammation resolution, vascularization, and osteogenesis	Level III	Relatively strong local targeting; can be combined with material platforms, and holds promise for achieving phased Immune regulation.	Release kinetics, delivery efficiency, batch consistency, and long-term safety are not yet clear; premature suppression of inflammation may impair antibacterial defense and necrotic tissue clearance; prolonged pro-repair bias may increase risk of fibrosis or abnormal tissue remodeling.	Lacks direct clinical trial data; overall still dominated by animal and materials science research.	(173–177)
Regulation via cells and Exosomes	MSCs, MSC-derived Exosomes	Regulates inflammation, vascularization, osteogenesis, and neurotrophic support through paracrine signaling via TSG-6, PGE2, TGF-β, IL-10, and miRNA	MSCs: Level II–III; Exosomes: Level III	Comprehensive multi-factor regulation, possessing potential for Immune regulation, pro-angiogenic, and pro-osteogenic effects.	Large variation in cell sources; non-uniform preparation processes and quality control; difficult to define effective components of Exosomes; potential risks of Immunogenicity, batch heterogeneity, excessive angiogenesis, ectopic ossification, or abnormal tissue remodeling.	MSCs have early clinical exploration in bone defects, non-union, or tissue repair; clinical evidence for Exosomes directly used in post-traumatic bone regeneration is still limited	(179–185)
Neuro-affinity and controlled- release biomaterials	Biomimetic periosteal materials, neuro-affinity scaffolds, NGF/CGRP/BMP-2 temporal release materials	Supports nerve ingrowth, neurovascular reconstruction, and osteogenic differentiation; modulates immune microenvironment through material structure.	Level I–III	Enables local delivery, spatial localization, and staged release; aids integrated nerve-vascular-bone reconstruction.	Complex system, high regulatory pathway and quality control requirements; dose matching issues for multi-factor release; overly rapid release or high local concentration may lead to pain sensitization, abnormal vascularization, ectopic bone formation, or an inflammatory response.	Some traditional bone repair materials and BMP-2 have clinical foundations; NGF/CGRP/immune factor neuro-immune-bone synergistic delivery is mostly pre-clinical evidence.	(187–193)
Conductive materials and electrical stimulation coupling	Conductive scaffolds, piezoelectric materials, exogenous electrical stimulation.	Mimics the electrophysiological microenvironment during bone repair, regulating stem cell fate, nerve growth, macrophage polarization, and immune-metabolic status.	Level I–III	Can match the electroactive characteristics of bone and nerves, potentially synergistically promoting nerve repair and bone regeneration	Stimulation intensity, frequency, duration, and material conductivity stability need standardization; improper parameters may lead to abnormal nerve excitation, local tissue irritation, inflammatory response, or adverse material interface reactions.	Traditional electrical stimulation has some clinical application foundation in delayed fracture healing or non-union; conductive scaffolds combined with neuro-immune regulatory factors are still mainly in animal studies.	(195–199)
Neuromodulation techniques	TENS, VNS	Regulates specific neural pathways and the cholinergic anti-inflammatory pathway, inhibiting excessive pro-inflammatory responses and promoting transition to a controlled reparative state.	Level II–III	Non-pharmacological intervention; TENS is non-invasive and easy to operate; VNS has a relatively clear cholinergic anti-inflammatory mechanism basis.	Specific efficacy for bone regeneration and optimal parameters remain unclear; VNS is invasive and device-dependent, and may affect heart rate, blood pressure, and autonomic nerve function.	There is clinical experience with TENS/VNS in pain management, rehabilitation, or related inflammation regulation, but direct clinical evidence for bone regeneration is insufficient.	(202–206)
Ultrasound regulation	LIPUS, Focused ultrasound	Promotes cell proliferation, migration, and osteogenic differentiation; Focused ultrasound may enable precise regulation of specific nerve bundles or local neural networks.	LIPUS: Level I–II; Focused ultrasound: Level III–IV.	Non-invasive, good spatial targeting capability; LIPUS has a certain research foundation in fracture healing.	Efficacy is inconsistent across different indications; acoustic intensity, frequency, pulse mode, depth of action, and treatment sessions need standardization; improper parameters may lead to thermal effects, excessive mechanical stress, or off-target tissue reactions.	LIPUS has clinical research and application foundation related to fracture healing; Focused ultrasound for precise neuro-immune axis regulation of bone regeneration is still mainly pre-clinical or theoretical exploration.	(208–211)

## 8 Current challenges and future directions

### 8.1 Heterogeneity and complexity of neuro-immune axis effects

The regulation of bone regeneration by the neuro-immune axis is not a uniform process but is highly dependent on anatomical site, injury type, and host status. Different bone tissues exhibit variations in neural innervation density, neural type composition, and local immune microenvironment. Thus, the same neuro-immune regulatory mechanisms may show different effect intensities and dominant pathways in long bones, jawbones, or vertebrae. This implies that mechanistic insights obtained from a specific bone tissue cannot be simply extrapolated to all bone regeneration scenarios. Differences in trauma type also profoundly affect the operational dynamics of the neuro-immune axis (212, 213). Closed fractures, open fractures with infection, and large segmental bone defects differ significantly in their profiles of DAMP/PAMP release, extent of soft tissue damage, scope of nerve injury, and stability of the local microenvironment. Consequently, the mode of inflammation initiation, neurogenic inflammation intensity, and inflammatory switch trajectory vary considerably (214). In milder injuries, neuro-immune responses may more easily maintain orderly activation and promote repair; whereas in severe trauma, infection, or nerve bundle transection, this axis is more prone to sustained amplification or dysregulation (215). Patient heterogeneity is a critical factor influencing the translation of related interventions. Age, diabetes, osteoporosis, infection status, smoking, peripheral neuropathy, soft tissue injury severity, fracture site, and fixation method can all modulate inflammatory response intensity, nerve regeneration capacity, angiogenesis levels, and osteogenic potential (216, 217). Thus, a single intervention strategy is unlikely to be suitable for all bone injury patients. Future research requires stratified designs based on pathological stage, injury characteristics, and host risk factors, rather than treating the neuro-immune axis as a static, universally applicable regulatory framework.

### 8.2 Differences in neuro-immune-bone regulation between animal models and human bone repair and translational limitations

Most current mechanistic evidence on neuro-immune axis regulation of bone regeneration still primarily comes from animal experiments, especially young, healthy rodent models. While these models have clear advantages in analyzing inflammatory switches, macrophage polarization, and neural signaling regulation, significant gaps remain compared to real clinical populations (218). The true translational challenge lies not in whether repair-promoting effects can be observed in ideal models, but in whether these effects can be stably reproduced in complex patient groups (219). First, animals and humans differ in bone structure, bone remodeling speed, and mechanical environment. Rodent bones are smaller, with faster callus formation and bone bridging, and their loading patterns, bone remodeling cycles, and internal fixation environments differ from human long bone fractures or large segmental defects (220, 221). Therefore, rapid callus formation, increased mineralization, or bone bridging observed in animal models cannot be directly equated with human clinical bone healing quality and long-term functional recovery.

Second, neural innervation and neuropeptide signaling also exhibit potential species differences. Animals and humans may not be entirely consistent in periosteal, bone marrow cavity, and perivascular neural innervation density, the ratio of sensory to sympathetic nerves, and the expression patterns of CGRP, SP, NE, and their receptors. This means that neuropeptide-induced pro-angiogenic, pro-osteogenic, or immunomodulatory effects observed in animal experiments cannot be simply extrapolated to human bone repair. Similarly, immune responses and inflammatory switches are species-specific. Macrophage polarization, neutrophil clearance, T cell involvement, and inflammation resolution speed in mice are not identical to the post-traumatic bone repair microenvironment in humans. M1/M2 transitions are relatively easy to observe and intervene in animal models but are often more complex in human fracture environments, influenced by factors such as age, infection, metabolic status, medication use, and local soft tissue injury severity (222, 223).

The complexity of real clinical trauma further widens the translational gap between animal models and human disease. Commonly used animal models mostly involve standardized fractures, bone defects, denervation, or single-factor interventions, whereas clinical patients often present with open injuries, severe soft tissue damage, infection, osteoporosis, diabetes, differences in fixation methods, rehabilitation compliance, and use of analgesics or anti-inflammatory drugs (224, 225). These factors can alter nerve regeneration, immune cell response thresholds, angiogenic capacity, and osteogenic potential. Especially in the context of aging, diabetes, osteoporosis, or pre-existing neuropathy, decreased nerve reinnervation capacity, abnormal neuropeptide release, persistent inflammation, and reduced bone marrow mesenchymal stem cell function may render the efficacy of interventions observed in healthy animals less certain in clinical settings (226).

Additionally, endpoint assessments in animal studies and clinical trials do not fully correspond. Animal studies often use histology, micro-CT, mechanical testing, gait, weight-bearing, or pain behavior as surrogate endpoints, while human clinical trials prioritize pain scores, weight-bearing capacity, joint range of motion, muscle strength, quality of life, work ability, and long-term functional recovery. If improvements in neuro-immune regulation observed in animals cannot be further translated into human pain relief, motor function improvement, and enhanced long-term bone healing quality, their clinical significance remains limited. Furthermore, clinical practice currently lacks real-time monitoring indicators that can dynamically reflect local neural activity, immune cell status, inflammatory switch progression, and nerve reinnervation quality (227). Therefore, the true gap from animal to clinic lies not only in the insufficient human relevance of current models but also in the difficulty of precisely capturing dynamic changes in the neuro-immune-bone axis in clinical settings to the same extent as in experimental systems.

Thus, future research should adopt models and validation pathways closer to clinical scenarios. On one hand, greater emphasis should be placed on developing models of aging, osteoporosis, diabetes, infection, open injuries, large segmental defects, or combined nerve injury, and large animal models appropriately introduced to more realistically simulate the mechanical environment, immune responses, and neural innervation characteristics of human bone repair. On the other hand, standardized structural and functional endpoints should be established, combining imaging, biomechanics, pain behavior, weight-bearing capacity, and motor recovery indicators. Ultimately, through human clinical samples, prospective cohorts, and mechanism-oriented clinical studies, future efforts must focus on validating whether the neuro-immune-bone regulatory mechanisms discovered in animal experiments truly apply to human post-traumatic bone regeneration.

### 8.3 Need for multi-omics and spatiotemporal dynamic analysis technologies

The key to this field in the future is not to add more descriptive studies but to use spatiotemporal multi-omics technologies to capture the dynamic dialogue within the neuro-immune-bone unit. Traditional histological and molecular biology methods provide static information but are limited in their ability to elucidate how neural signals, immune cell states, and osteogenic programs interact at different time points and spatial regions during bone regeneration. In contrast, single-cell RNA sequencing, spatial transcriptomics, and high-dimensional protein analysis technologies offer new possibilities for dissecting this dynamic network (228, 229). For instance, single-cell resolution analysis can delineate state transitions in macrophages, T cells, and osteogenic precursor cells across distinct stages of bone regeneration, thereby clarifying which immune subsets drive the inflammatory switch and identifying the specific neural-related receptors or ligands involved (230). Spatial transcriptomics can then contextualize these findings within the callus, periosteum, perivascular, and innervation regions, revealing the specific cellular crosstalk and its spatial localization (231). Therefore, what truly matters in the future is not to continue accumulating more facts about “which cells and which molecules are present,” but to use methods with higher spatiotemporal resolution to explain how the neuro-immune-bone regeneration network completes the programmed switch from inflammation initiation to repair and reconstruction at different stages (232). On this basis, if clinically compatible dynamic monitoring methods, such as fluid biomarkers, molecular imaging, or local microenvironment monitoring technologies, can be further developed, it may be possible to gradually translate dynamic insights from basic research into clinically evaluable indicators. However, the value of such technologies should first serve mechanism clarification and key node identification, rather than simply expanding into a technical checklist.

### 8.4 Building integrated predictive and intervention models

The complexity of the neuro-immune axis suggests that future bone regeneration research needs to shift from single-indicator judgments to more integrated predictive and intervention frameworks. This framework should link imaging information, inflammatory status, neural-related biomarkers, and functional recovery indicators to identify high-risk repair trajectories earlier and provide a basis for stratified interventions (233). At the same time, the model should not only evaluate pro-healing effects but also incorporate safety endpoints such as pain sensitization, abnormal nerve sprouting, infection risk, fibrosis, heterotopic ossification, excessive immune suppression, and systemic autonomic side effects. Future translational research should also incorporate dose, timing, safety, off-target effects, patient heterogeneity, and clinical feasibility into the same evaluation framework. Candidate interventions need not only to demonstrate improvement in bone regeneration-related mechanistic indicators but also to clarify the optimal intervention window, dose-response relationship, local and systemic adverse effects, applicable patient subgroups, operational convenience, cost, and regulatory feasibility. For example, if some patients simultaneously exhibit persistent high inflammatory burden, abnormal neuropeptide levels, and delayed functional recovery, this may indicate that their neuro-immune axis has deviated from the normal repair trajectory, requiring earlier and more targeted intervention (234).

Regarding therapeutic strategies, the significance of an integrated model lies in helping to determine the optimal prioritization of intervention targets for different patients and stages (235). For some patients, the focus may be on mitigating excessive early inflammation; for others, it may be on promoting later nerve reinnervation, breaking the pain-disuse cycle, or optimizing functional recovery (236, 237). Therefore, future research should not merely add more intervention methods but gradually establish a translational pathway from mechanism identification and risk stratification to stage-specific intervention selection. This framework also helps avoid simplistic strategies such as “promoting repair at all costs” or “suppressing inflammation at all costs,” incorporating efficacy assessment and adverse reaction monitoring into the research design simultaneously.

## 9 Conclusion

Post-traumatic bone regeneration is not merely a process of bone tissue repair, but a coupling process involving the nervous, immune, and bone remodeling systems. Its key lies not in simply amplifying or suppressing inflammation, but in achieving a timely switch from the early clearance program to the later repair program of inflammation through the neuro-immune axis. Neuropeptides, neurotransmitters, and related immune effector molecules orchestrate the initiation of inflammation, macrophage polarization, angiogenesis, and osteogenic regulation, ultimately determining the efficiency and quality of bone regeneration. The clinical significance of this axis extends beyond influencing bony healing to profoundly impacting pain control and functional rehabilitation. Dysregulation of the neuro-immune axis may lead to persistent inflammation, impaired macrophage polarization, insufficient reinnervation, and amplified neurogenic inflammation, thereby increasing the risk of nonunion, chronic pain, and motor dysfunction. Therefore, the evaluation and intervention of trauma repair should not be limited to structural bone connection but should also focus on functional endpoints such as pain, weight-bearing, joint range of motion, and motor recovery, promoting equal emphasis on structural repair and functional rehabilitation. Future intervention strategies should not only remain confined to the “hardware” level of supplementing scaffolds, materials, or growth factors but should also prioritize the regulation of the “software” level, such as inflammation switching, reinnervation, and local cellular communication networks. Further development in this field depends on the deep integration of neuroscience, osteoimmunology, biomaterials science, and regenerative medicine, and relies on spatiotemporal multi-omics and dynamic monitoring technologies to more precisely identify key regulatory nodes and appropriate intervention time windows, thereby promoting post-traumatic bone regeneration from simple structural repair to higher-quality functional reconstruction.
